# Uncovering abnormal gray and white matter connectivity patterns in Alzheimer’s disease spectrum: a dynamic graph theory analysis for early detection

**DOI:** 10.3389/fnagi.2025.1589018

**Published:** 2025-07-22

**Authors:** Juanjuan Jiang, Tao Kang, Ronghua Ling, Yingqian Liu, Jiuai Sun, Yiming Li, Xiaoou Li, Hui Yang, Bingcang Huang, the Alzheimer’s Disease Neuroimaging Initiative

**Affiliations:** ^1^School of Medical Imaging, Shanghai University of Medicine and Health Science, Shanghai, China; ^2^AVIC Huadong Optoelectronics (Shanghai) Co., Ltd, Shanghai, China; ^3^School of Electrical Engineering, Shandong University of Aeronautics, Binzhou, China; ^4^College of Medical Instrument, Shanghai University of Medicine & Health Sciences, Shanghai, China; ^5^Department of Radiology, Shanghai Pudong New Area Gongli Hospital, Shanghai, China

**Keywords:** brain imaging, dynamic network, gray and white matter, functional network, Alzheimer’s disease

## Abstract

**Background:**

Alzheimer’s disease (AD) requires early intervention at preclinical stages like subjective memory complaints (SMC). Traditional static brain network analyses lack sensitivity to detect early functional disruptions in SMC. This study aimed to improve preclinical AD stratification by integrating dynamic gray-white matter functional connectivity (DFC) and machine learning.

**Methods:**

Using multi-cohort ADNI data [*N* = 1,415 participants across cognitive normal[CN], SMC, and cognitive impairment [CI]groups],dynamic functional networks were constructed via sliding-window analysis (20–50 TR windows, 98% overlap) of 200 gray matter (Schaefer atlas) and 128 data-driven white matter nodes. DFC metrics (standard deviation of Fisher z-transformed correlations) were used to identify group differences and classify AD spectrum stages. Support vector machine (SVM) models were trained to differentiate CN/SMC/CI, with subgroup analyses in Aβ + and APOE E4 + populations.

**Results:**

DFC with short sliding windows (20–50 TRs, 98% overlap) demonstrated greater sensitivity than SFC in detecting early functional disruptions in gray-white matter networks, identifying 34 CN-SMC [*p* < 0.05, e.g., ventral attention network (VAN)-white matter 2 (WM2) via Gau20-DFC], 44 CN-CI (*p* < 0.001), and 49 SMC-CI (*p* < 0.01) differential connections. Key early abnormalities were identified in the anterior cingulate network (WM4) and sensorimotor network (WM5), with WM4-WM5 disconnections in Aβ + subgroups strongly correlated with Aβ deposition and APOE ε4 genotype. Dynamic graph theory models using SVM achieved superior AD spectrum classification (ADNI2/3 AUCs: 0.85–0.92 vs. static 0.77–0.87), particularly in Aβ + subgroups (ΔAUC = 0.15 for SMC+/CI + discrimination, *p* < 0.001), with the VAN-WM2 feature in short-window DFC strongly correlating with cognitive scales (MMSE: *r* = 0.40, *p* < 10^−11^; CDR-SB: *r* = −0.41, *p* < 10^−12^). Window function type (e.g., Gau20 for early changes, Ham50 for late stability) and data sampling points influenced sensitivity, with short windows optimizing early detection and long windows capturing late-stage network degeneration. These findings establish dynamic gray-white matter connectivity, particularly WM4-WM5 disruptions and VAN-WM2/DMN-WM8 features, as sensitive preclinical AD biomarkers enabled by machine learning for early SMC stratification.

**Conclusion:**

This study confirms that dynamic gray-white matter connectivity serves as a sensitive biomarker for preclinical Alzheimer’s disease. The WM4-WM5 disruption hub and machine learning framework provide effective tools for early stratification of SMC, facilitating timely intervention within the disease’s therapeutic window.

## 1 Introduction

Alzheimer’s disease (AD) is a prevalent neurodegenerative disorder with a progressive, irreversible course, significantly threatening elderly physical and mental health (Singh et al., 2016; Østerhus, 2020). Early intervention is critical due to AD’s irreversible damage and progression (Rasmussen and Langerman, 2019). Subjective memory complaints (SMC) represent a potential preclinical AD stage, where identification and treatment may delay progression (Choe et al., 2018; Warren et al., 2022; Amariglio et al., 2012). Amyloid-*β* (Aβ) and apolipoprotein E (APOE) E4 status are incorporated into AD spectrum classification criteria, playing key roles in early diagnosis (Schmechel, 1995; Castellano et al., 2011). Resting-state functional MRI (rs-fMRI)-based brain network analysis, effective for characterizing complex network topology, is a valuable early AD diagnostic tool (Supekar et al., 2008). Prior studies report altered functional network topology in AD and preclinical stages, such as declining node clustering and modularity with increasing Clinical Dementia Rating Scale (CDR) scores, indicating large-scale connectivity disruptions (Brier et al., 2012; Zhao et al., 2012). Global functional connectivity assessments show reduced efficiency and clustering coefficients in AD, particularly in bilateral hippocampi (Supekar et al., 2008; Yang et al., 2018).

Current dynamic functional magnetic resonance imaging (fMRI) methods often use fixed sliding windows with strong prior assumptions, limiting generalizability across ethnic groups and scanners (Qin et al., 2024; Liuzzi et al., 2019; Filippi et al., 2019). High individual heterogeneity in SMC further challenges existing approaches (Warren et al., 2022). Machine learning advancements have improved fMRI-based AD diagnosis by integrating detailed image features and optimizing classification (Klöppel et al., 2013; Khan et al., 2021; Jia and Lao, 2022). Unlike static functional connectivity (SFC), which assumes constant networks, dynamic functional connectivity (DFC) captures transient fluctuations, enhancing sensitivity to early pathology (Supekar et al., 2008) (Córdova-Palomera, 2017). For example, 20-TR sliding-window DFC detects subtle A𝛽-related disruptions in the posterior default mode network (DMN) and white matter tracts, correlating with amyloid PET load (Palmqvist, 2017; Ali et al., 2023; Reijmer et al., 2016).

Dynamic graph theory metrics (e.g., modularity, path length variability) reflect network integration/segregation dynamics (Ghanbari et al., 2023; Liao et al., 2018; Xia et al., 2010), outperforming static metrics in early AD classification (Chen et al., 2021; Yang et al., 2022; Hu et al., 2020). These spatiotemporal features improve machine learning models; e.g., Karim et al. achieved 92% AD classification accuracy using rs-fMRI and graph metrics with SVM (Karim et al., 2024), while Hojjati et al. reported 89–97% accuracy for MCI conversion prediction using multimodal MRI (Lin et al., 2023). Chen et al. distinguished SMC from controls with 79.23% accuracy via functional connectivity and graph parameters (Hao et al., 2019).

**Figure 1:**
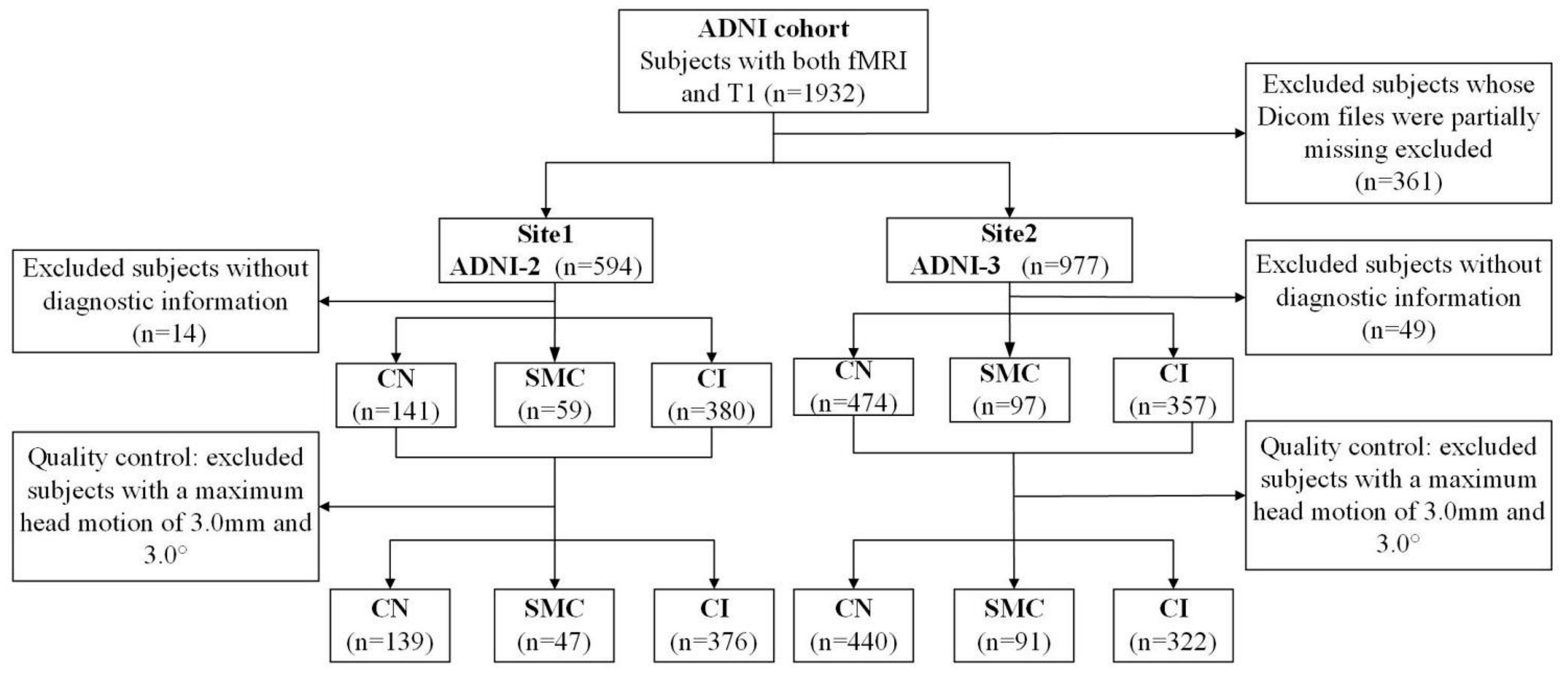
The flowchart of all participants in this study.

**Figure 2:**
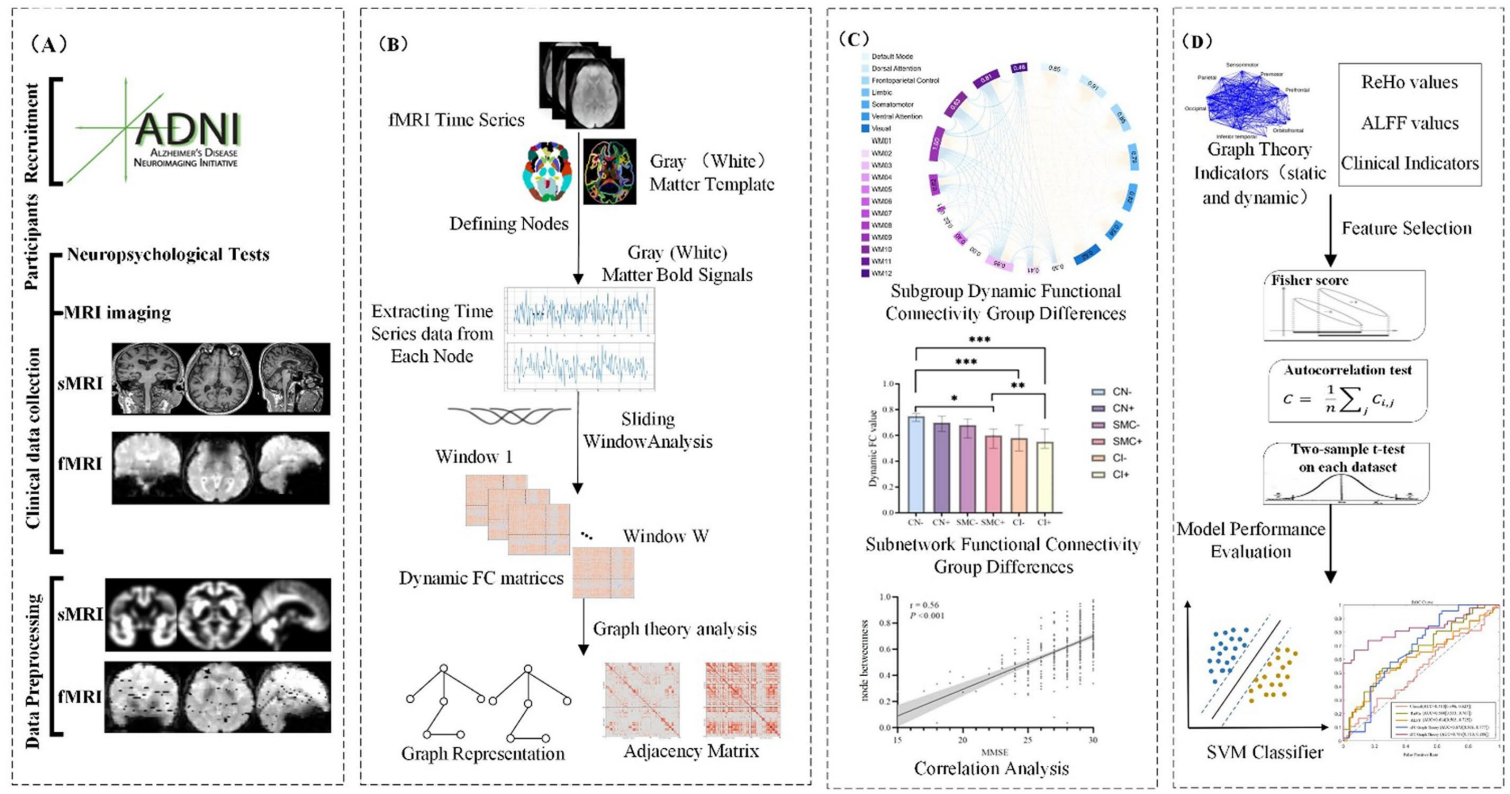
The workflow of this study.

**Figure 3:**
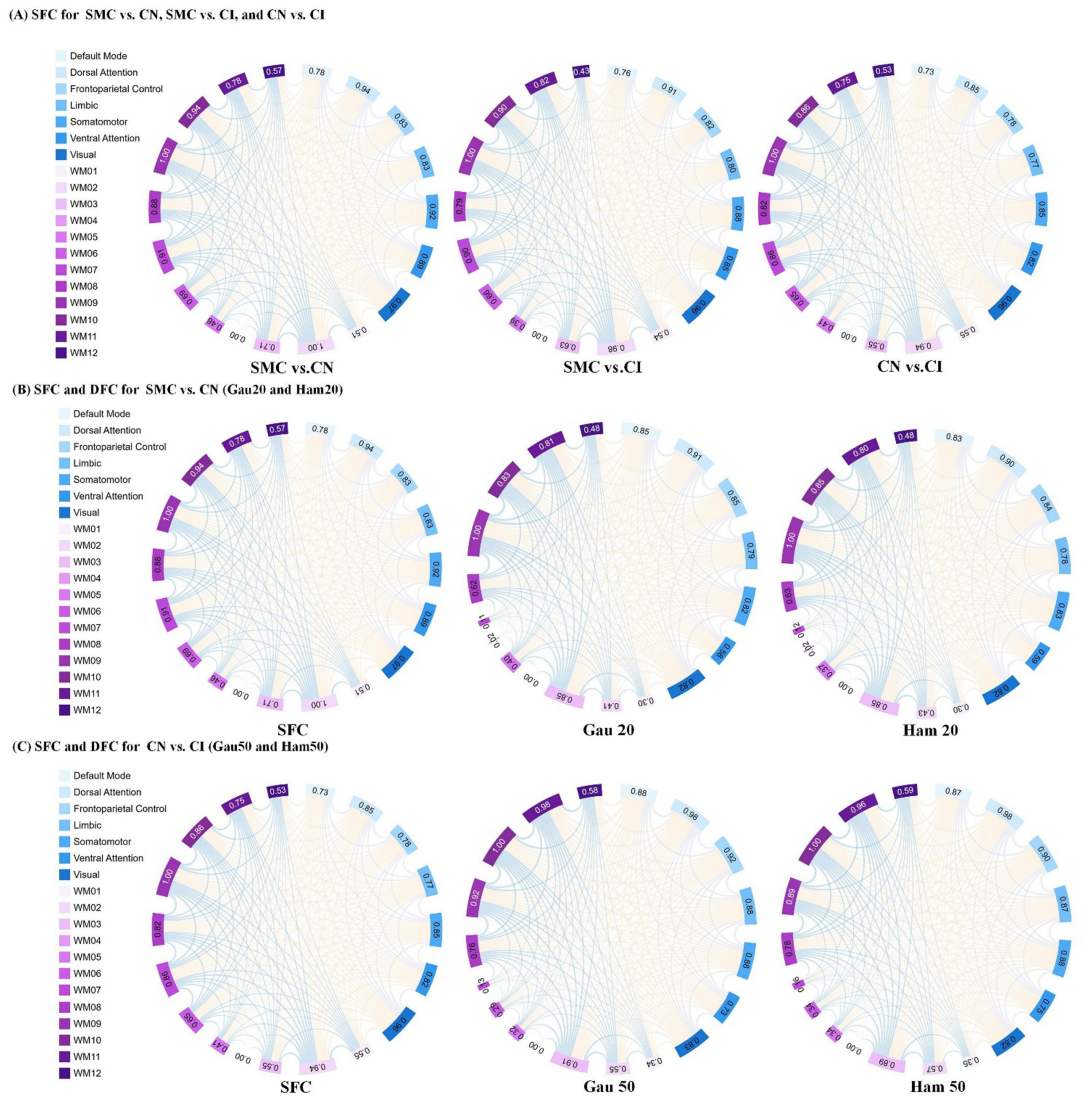
Presents an analysis of inter-group differences based on static and dynamic brain gray and white matter functional networks, visualized as chord diagrams that illustrate functional connectivity (FC) pairs showing group differences. At the ROI level, FC pairs with group differences were identified using two - sample *t*-tests. ROIs were categorized according to 7 gray matter sub- networks and 12 white matter sub-networks. For each sub-network, the sum of connection strengths exhibiting differences was computed. The circumference of each chord diagram is segmented into 7 parts, where the length of each segment represents the connection strength of the corresponding sub - network. The thickness of the connecting lines indicates the strength of connectivity between brain regions. Panel **(A)** displays SFC (static functional connectivity) for the group comparisons of SMC vs. CN, SMC vs. CI, and CN vs. CI. Panel **(B)** shows both SFC and DFC (dynamic functional connectivity) with Gaussian window of 20 s (Gau20) and Hamming window of 20 s (Ham20) for the SMC vs. CN comparison. Panel **(C)** presents SFC and DFC with Gaussian window of 50 s (Gau50) and Hamming window of 50 s (Ham50) for the CN vs. CI comparison.

**Figure 4:**
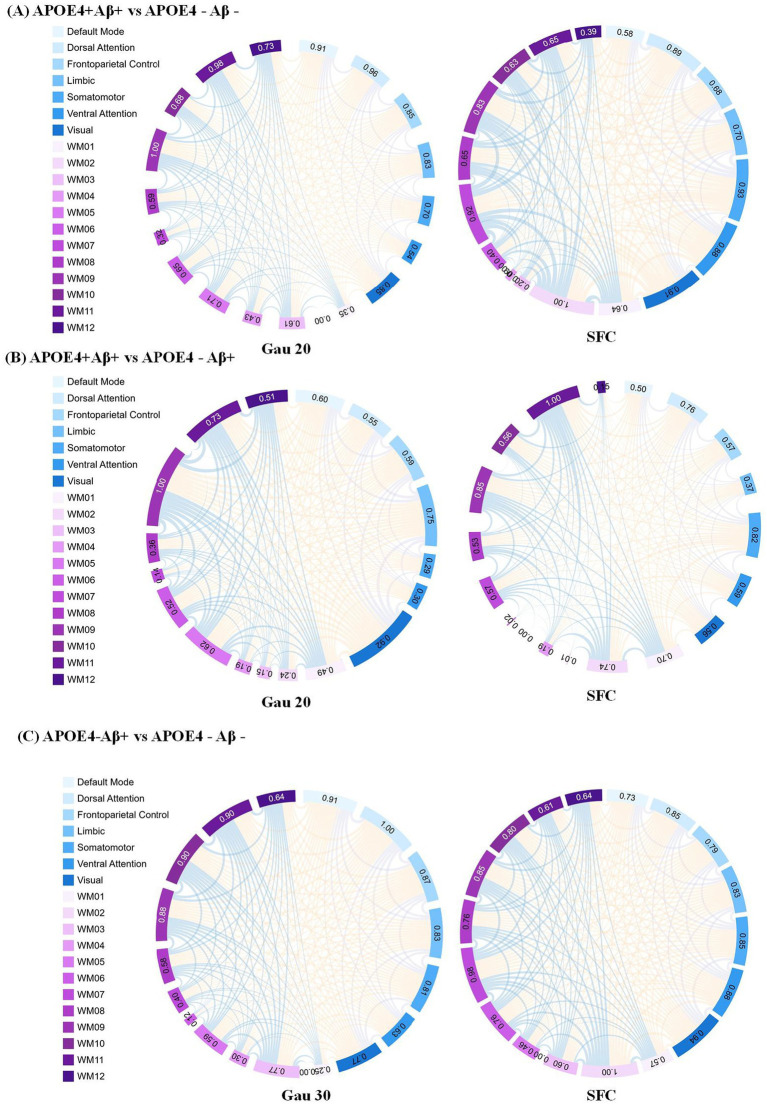
Static functional connectivity (SFC) as a complement to dynamic network analysis in APOE4 × A*β* subgroups **(A)** SFC differences between APOE4 + Aβ + and APOE4 − Aβ − groups under the Gau20 window (20 s Gaussian). Thicker edges reflect stable connectivity disruptions, which persist despite transient dynamic changes (e.g., Ham20 window DFC). This highlights SFC’role in capturing long-term network damage from combined APOE4 and Aβ risk factors. **(B)** SFC differences between APOE4 + Aβ + and APOE4 − Aβ + groups under the Gau20 window. Static connectivity patterns here contrast with dynamic reverse regulation (e.g., Ham20 DFC), suggesting APOE4 × Aβ interactions modulate both stable and transient network states. **(C)** SFC differences between APOE4 − Aβ + and APOE4 − Aβ − groups under the Gau30 window (30 s Gaussian). Subtle static changes in APOE4-deficient subgroups align with dynamic instability (e.g., Ham30 DFC), supporting Aβ’s independent impact on both static and dynamic networks. Subnetworks include 7 gray-matter networks (e.g., Default Mode) and 12 white-matter tracts (WM01–WM12). SFC = Static Functional Connectivity; DFC = Dynamic Functional Connectivity; Gau20/30 = 20/30 s Gaussian windows.

Despite progress, SMC-stage research remains limited (Sajid et al., 2024). Therefore, developing a computer-aided diagnosis model for SMC using dynamic brain network graph theory parameters and machine learning methods holds significant clinical value. Building on these prior findings, our study addresses this gap by integrating dynamic gray-white matter connectivity and machine learning. Using multi-cohort ADNI data, we hypothesized that short-window 20 TR DFC metrics would surpass SFC in detecting early SMC disruptions. Combining data-driven white matter parcellation with dynamic graph theory, we aimed to identify A𝛽/APOE E4-linked connectivity hubs (e.g., anterior cingulate WM4, sensorimotor WM5) and develop an SVM-based classifier for CN/SMC/CI discrimination, with subgroup analyses in A𝛽+ and APOE E4 + populations. Our findings seek to establish dynamic brain networks as reliable tools for preclinical AD stratification and timely SMC intervention (Figures 1–4).

## 2 Materials and methods

### 2.1 Participants

Data used in the preparation of this article were obtained from the Alzheimer’s disease Neuroimaging Initiative (ADNI) database (adni.loni.usc.edu). The ADNI was launched in 2003 as a public-private partnership, led by Principal Investigator Michael W. Weiner, MD. The primary goal of ADNI has been to test whether serial MRI, positron emission tomography, other biological markers, and clinical and neuropsychological assessment can be combined to measure the progression of MCI and early AD. This study utilized neuroimaging and clinical data from the Alzheimer’s disease Neuroimaging Initiative phases ADNI-2 and ADNI-3, with cohort partitioning based on fMRI temporal acquisition protocols. The combined sample comprised 1,415 participants: 562 from ADNI-2 (139 cognitive normal [CN], 47 SMC, 376 cognitive impairment [MCI/AD]) and 853 from ADNI-3 (440 CN, 91 SMC, 322 cognitive impairment). Inclusion criteria required: (1) complete T1-MRI and resting-state fMRI scans (ADNI-3 subjects had synchronized multimodal imaging); (2) APOE genotype and CSF Aβ42 data (for Aβ + classification: Aβ42 < 980 pg./mL); (3) stringent motion control (framewise displacement <0.5 mm). Diagnostic categorization followed ADNI criteria: CN (CDR = 0, MMSE ≥ 26, no cognitive deficits), SMC (self-reported memory decline >6 months with intact neuropsychological scores), MCI (CDR = 0.5, objective memory impairment >1.5 SD), and AD (CDR ≥ 1, MMSE < 24, NIA-AA criteria). The APOE4+/Aβ + subgroup required ε4 allele positivity and biomarker-confirmed amyloid pathology (Centiloid > 20). Ethical approval for all studies was obtained from the respective institutional review committees, and written informed consent was obtained from each participant.

### 2.2 fMRI imaging scanning and preprocessing

Structural and functional MRI data were preprocessed using the standardized pipeline implemented in DPARSF.^1^ Initial fMRI volumes (*n* = 10) were discarded to ensure magnetization equilibrium, followed by slice-timing correction and rigid-body motion realignment. Participants demonstrating excessive head movement (>3.0 mm translation or >3.0° rotation across any axis) were systematically excluded. Functional images were co-registered to individual T1-weighted anatomical scans and normalized to MNI space (3 × 3 × 3 mm^3^ isotropic resolution). Subsequent preprocessing included: (1) nuisance regression (24-parameter motion profiles, white matter/CSF signals) to mitigate physiological artifacts; (2) temporal bandpass filtering (0.01–0.08 Hz) for low-frequency drift removal; and (3) spatial smoothing (6 mm FWHM Gaussian kernel). Given the multi-site nature of ADNI-2/3 data, we addressed inherent heterogeneity arising from inter-scanner variability, acquisition parameter differences, and demographic confounders (age/gender distribution) through ComBat harmonization. This empirical Bayes framework, originally developed for genomic data integration, effectively removes site-specific technical biases while preserving biological variance, as demonstrated in recent neuroimaging applications (Fortin et al., 2018). Full acquisition parameter specifications across scanners are provided in online website.^2^

### 2.3 Dynamic brain network construction

This study constructs dynamic gray-white matter functional connectivity networks based on fMRI data, integrating gray matter and white matter information to investigate the dynamic interactions within the brain. The detailed process is as follows:

#### 2.3.1 Node definition

Cortical gray matter nodes were defined using the Schaefer 200 atlas (Schaefer et al., 2018), partitioning the cerebral cortex into 200 functionally homogeneous regions reorganized into seven subnetworks: default mode, visual, somatomotor, limbic, frontoparietal control, dorsal attention, and salience/ventral attention. These include AD-susceptible regions like the posterior cingulate cortex and medial prefrontal cortex. For white matter, a data-driven clustering approach (Peer et al., 2017) was applied: a group-averaged white matter mask was created, and K-means clustering (2–22 clusters) identified 128 white matter regions across 12 functional networks, validated via four subsets with a Dice coefficient >0.85.

#### 2.3.2 Functional network construction

Average blood oxygen level-dependent (BOLD) signals were extracted from gray/white matter regions, and Pearson correlations were computed for all node pairs (gray-gray, white-white, gray-white) to construct 200 × 200 gray matter, 128 × 128 white matter, and 328 × 328 gray-white matter functional connectivity matrices.

#### 2.3.3 DFC network construction

To comprehensively assess the dynamic changes in brain functional networks, we employed a sliding window approach to construct DFC networks, quantifying the functional connections between gray matter, white matter, and gray-white matter regions.

- *Sliding window selection*: 20, 30, and 50 TR windows (1 TR step, 98% overlap) were tested. The optimal 30 TR window balanced dynamic sensitivity and noise control, with permutation tests correcting autocorrelation.
- *Window function selection*: Hamming (reducing edge effects) and Gaussian (noise resistance) windows were used to comprehensively evaluate connectivity dynamics.
- *Dynamic functional connectivity quantification*:

For each sliding window, Pearson correlation coefficients were calculated between the time series of gray and white matter nodes. The formula is:

$$r=\frac{\sum (x_{i}-\bar{x})(y_{i}-\bar{y})}{\sqrt{\sum {\left(x_{i}-\bar{x}\right)}^{2}\sum {\left(y_{i}-\bar{y}\right)}^{2}}}$$

where $x_{i}$ and $y_{i}$ are the BOLD signals of two nodes, and $\bar{x}$ and $\bar{y}$ are their respective means.

- *Fisher z-transformation*: To normalize the distribution of correlation coefficients for accurate statistical comparisons, Fisher’s r -to-*z* transformation was applied:
$$z_{x,y}=\frac{1}{2}\ln(\frac{1+r_{x,y}}{1-r_{x,y}})$$

where $r_{x,y}$ denotes the correlation coefficient between node x and node y, $z_{x,y}$ denotes the transformed z-score.

- *Dynamic variability metric*: The dynamic variability of functional connectivity was quantified by calculating the standard deviation (SD) of the z-transformed correlation values across all windows:

$$\text{Dynamic Variability}=\mathrm{SD}(z_{\text{values}})$$

This metric reflects the amplitude of connectivity strength fluctuations over time.

### 2.4 Machine learning analysis

To develop a robust classification model for distinguishing between different cognitive stages (CN, SMC, and CI) using DFC metrics, we employed a comprehensive machine learning pipeline with Support Vector Machine (SVM) as the classification model. The detailed analysis pipeline is described as follows:

#### 2.4.1 Feature extraction

##### 2.4.1.1 Dynamic connectivity metrics

We extracted dynamic connectivity metrics from the sliding-window analysis of the gray-white matter functional networks. These metrics included the standard deviation of Fisher z-transformed correlation values across all windows, reflecting the temporal variability of functional connections. This process generated a comprehensive set of features representing the dynamic interactions between gray matter and white matter regions.

##### 2.4.1.2 Feature set size

The initial feature set included over 21,000 dynamic connectivity metrics derived from the 328 × 328 gray-white matter network. Each feature represented the dynamic variability of a specific functional connection between two nodes (gray-gray, white-white, and gray-white).

#### 2.4.2 Feature selection

##### 2.4.2.1 LASSO regularization

To identify the most relevant features for classification and reduce dimensionality, we applied LASSO (Least Absolute Shrinkage and Selection Operator) regression. LASSO helped in selecting a subset of features that contributed most significantly to the classification performance while minimizing overfitting. The optimal regularization parameter was determined through cross-validation. Ultimately, LASSO selected 1,200 features that were most relevant for distinguishing between the different cognitive stages.

#### 2.4.3 Clinical relevance validation of selected features

To ensure the biological and clinical interpretability of the selected features, Pearson correlation analyses were performed between dynamic connectivity metrics (e.g., VAN-WM2 Gau20 std) and neuropsychological scales (MMSE, CDR-SB). This step aimed to assess whether the features aligned.

#### 2.4.4 Model training and validation

##### 2.4.4.1 Cross-validation

We employed 10-fold cross-validation to train and validate the SVM model, a robust approach to assess generalizability across the ADNI multi-cohort dataset (1,415 participants). The dataset was partitioned into 10 subsets, with 9 subsets used for training and 1 for validation in each iteration, ensuring each sample was validated once. This method minimized selection bias and provided reliable performance estimates, particularly for detecting subtle differences in the SMC stage.

#### 2.4.5 Performance evaluation

##### 2.4.5.1 Evaluation metrics

Model performance was quantified using accuracy, sensitivity, specificity, and AUC-ROC, with a focus on differentiating early (SMC) vs. late (CI) stages. For example, in Aβ + subgroups, the dynamic model achieved an AUC of 0.92 for SMC + vs. CI + discrimination (ΔAUC = 0.15 vs. static model, *p* < 0.001; Table 1), highlighting its sensitivity to early pathology.

**Table 1:** Classification results of Aβ subgroup and ApoE ε4 gene subgroup.

	Aβ subgroup			ApoE ε4 subgroup		
Classification features	Accuracy (%)	Sensitivity (%)	Specificity (%)	Accuracy (%)	Sensitivity (%)	Specificity (%)
Clinical	55.47 ± 12.11	56.25 ± 11.46	54.07 ± 13.93	57.18 ± 8.97	51.33 ± 10.98	59.15 ± 10.54
ReHo	64.51 ± 2.03	66.92 ± 4.23	60.17 ± 8.10	68.55 ± 4.35	68.67 ± 5.03	68.51 ± 5.54
ALFF	66.72 ± 2.98	70.69 ± 5.68	59.59 ± 4.30	69.57 ± 2.71	66.07 ± 8.27	70.75 ± 5.23
Static graph theory	78.25 ± 6.73	78.77 ± 9.45	79.31 ± 9.48	77.91 ± 6.26	77.33 ± 8.36	80.11 ± 8.02
Dynamic graph theory	**83.63 ± 4.02**	**83.25 ± 4.71**	**84.31 ± 6.9**	**85.16 ± 3.96**	**82.26 ± 6.1**	**86.14 ± 4.63**

Data are presented as mean ± standard deviation.

##### 2.4.5.2 Class imbalance handling

To address skewed class distributions (e.g., CI: SMC ratio ~8:1 in ADNI-2), SMOTE was applied to synthetically oversample minority classes during training. This improved the model’s ability to detect SMC cases, as evidenced by a 12% increase in F1 score for the SMC class (Supplementary Table S5). Confusion matrices confirmed balanced performance across CN/SMC/CI categories, with misclassification rates <15% in cross-validation.

### 2.5 Statistical analyses

In our study, one-way analysis of variance (ANOVA) was performed to analyze the demographic information, plasma biomarkers, and mean functional connectivity (FC) of subnetworks across different groups. To further explore differences between groups, Tukey’s multiple comparisons test was employed following the ANOVA. We applied two-sample t-tests to identify region of interest (ROI)-level FC pairs that exhibited differences between groups. The chi-square test was used to examine group differences for discrete variables, and the independent two-sample t-test was used to examine statistical differences between continuous variables. Significance level was set at *p* < 0.05. To establish the clinical relevance of identified dynamic connectivity features, Pearson correlation coefficients were calculated between dynamic metrics (e.g., standard deviation of Fisher z-transformed correlations for VAN-WM2, DMN-WM5, and other key connections across different window types) and clinical cognitive scales (MMSE, MoCA, and CDR-SB). A significance level of *p* < 0.05 was used, with Bonferroni correction applied to address multiple comparisons. These analyses were performed to evaluate whether temporal fluctuations in gray-white matter connectivity correlate with cognitive impairment severity. Receiver operating characteristic (ROC) curves were used to assess the ability to discriminate among different groups.

## 3 Results

### 3.1 Demographic and clinical characteristics

Table 2 summarizes the baseline characteristics of ADNI-2 and ADNI-3 cohorts. In ADNI-2, significant differences were observed between the SMC and CI groups in APOE ε4 allele frequency (34.0% vs. 56.7%, *p* < 0.001), Aβ positivity (29.8% vs. 21.5%, *p* = 0.032), MMSE scores (28.9 ± 1.1 vs. 25.7 ± 3.8, *p* < 0.001), and CDR-SB scores (0.21 ± 0.72 vs. 2.68 ± 2.18, *p* < 0.001). Similar trends were replicated in ADNI-3, with APOE ε4 prevalence (31.9% vs. 45.3%, *p* = 0.002) and MMSE scores (29.0 ± 1.2 vs. 25.9 ± 4.0, *p* < 0.001) differing significantly between SMC and CI groups. No significant age or gender differences were noted across cognitive stages (*p* > 0.05).

**Table 2:** Clinical and baseline demographic characteristics of subjects from ADNI.

ADNI-2 (*n* = 562)				ADNI-3 (*n* = 853)		
	CN *n* = 139	SMC *n* = 47	CI *n* = 376	CN *n* = 440	SMC *n* = 91	CI *n* = 322
Sex (M/F)	58/81	20/27	207/169	176/264	34/57	185/137
Age (years)	75.0 ± 6.3	72.6 ± 5.2	73.4 ± 7.2	73.2 ± 7.4	76.6 ± 5.5	76.4 ± 8.2
Education (years)	16.7 ± 2.1	16.9 ± 3.0	15.9 ± 2.6	16.8 ± 2.2	16.6 ± 2.4	15.7 ± 2.7
ApoE ε4 (−/+)	83/56	31/16	163/213	261/142	62/29	172/121
Aβ (−/+)	31/13	20/14	38/81	191/60	47/18	64/72
MMSE	29.0 ± 1.4	28.9 ± 1.1^a*^	25.7 ± 3.8^a*^	29.0 ± 1.8	29.0 ± 1.2^b*^	25.9 ± 4.0^b*^
MoCA	25.8 ± 2.25	26.1 ± 2.25^a*^	21.2 ± 4.9^a*^	26.2 ± 2.63	26.6 ± 2.8^b*^	20.8 ± 4.6^b*^
CDR-SB	0.09 ± 0.21	0.21 ± 0.72^a*^	2.68 ± 2.18^a*^	0.08 ± 0.27	0.39 ± 0.69^b*^	2.76 ± 2.74^b*^

All data are expressed as mean ± standard deviation. MMSE, Mini-mental State Examination; MoCA, Montreal cognitive assessment; CDR-SB, The sum of boxes of clinical dementia rating scale, CN Cognitively normal, SMC Subjective Memory Concerns, CI cognitive impairment, Aβ:β-amyloid; * indicates *p* < 0.05; ^a^indicates a significant statistical difference between the SMC group and the CI group in ADNI2. ^b^indicates a significant statistical difference between the SMC group and the CI group in ADNI3. Age, Education, MMSE, MoCA-B, CDR-SB performed a nonparametric rank sum test to compare differences in demographic and clinical characteristics between the training/validation and test groups under each label, i.e., CN, CI, and SMC; gender was tested by chi-square between the two groups under each label.

### 3.2 Stability and differential analysis of DFC and SFC across ADNI cohorts

Stability of functional connectivity (FC) networks across ADNI cohorts is presented in Supplementary Table S2. For cognitively normal (CN) groups, similarity coefficients of SFC were 0.769 (ADNI2 internal), 0.769 (ADNI3 internal), and 0.777 (ADNI2 vs. ADNI3). In subjective memory concern (SMC) groups, SFC coefficients ranged from 0.783–0.785. DFC showed higher consistency than SFC across all window types (Hamming/Gaussian, 20–50 TRs), with similarity coefficients spanning 0.862–0.933. The lowest DFC coefficient was 0.862 (Ham50, ADNI2 CN), while the highest was 0.933 (Gau20, ADNI2 vs. ADNI3 SMC), indicating stronger cross-cohort stability for short-window dynamic metrics.

Supplementary Table S3 and Table 3 (main text) show group-wise FC differences. In ADNI2 Site 3, SMC vs. CN revealed 30 differential DFC connections (Ham20) in ventral attention networks and white matter subnetworks (WM2/WM5), whereas CN vs. CI had 54 SFC connections in default mode and visual networks. In ADNI3 Site 1, CN vs. CI exhibited 118 SFC connections—significantly more than SMC vs. CN (2 connections)—reflecting progressive static network degradation in late stages.

**Table 3:** Subset-level static vs. dynamic FC in ADNI 3.

Differential connections (Pairs)		ADNI-3					
		Site1			Site2		
		SMC vs. CN	SMC vs. CI	CN vs. CI	SMC vs. CN	SMC vs. CI	CN vs. CI
SFC		2	54	118	2	22	94
DFC	Ham20	38	60	8	6	6	14
	Ham30	22	40	6	6	6	12
	Ham40	10	28	8	8	18	16
	Ham50	6	32	2	6	18	14
	Gau20	36	60	12	10	6	16
	Gau30	32	42	10	6	6	12
	Gau40	16	22	8	10	16	14
	Gau50	10	32	6	8	18	20

Ham20 refers to a Hamming window with a size of 20 s. Gau20 refers to a Gauss window with a size of 20 s. Similarly, Gau30, Gau40, and Gau50 represent Gauss windows with sizes of 30 s, 40 s, and 50 s accordingly. Site 1 and Site 2 differ in the number of fMRI time points, where Site 1 includes data with 187 time points, and Site 2 with 140 time points.

Subgroup analyses (Supplementary Table S4; Table 4) showed that within A*β* + groups, APOE4 + carriers had 70 differential DFC connections (Gau20) compared to 34 in APOE4– carriers. The APOE4 + Aβ + vs. APOE4–Aβ– comparison revealed 136 SFC connections in default mode and limbic networks, indicating synergistic genetic-pathological effects. Additionally, Table 5 further analyzes the independent impact of APOE4 in Aβ + and Aβ- populations. Results show that within the Aβ + group, the number of SFC differential connections between APOE4 + and APOE4- was 82 pairs, and the number of DFC (such as Gau20) differential connections was 70 pairs. In the Aβ- group, the number of SFC and DFC differential connections between APOE4 + and APOE4- decreased significantly (70 pairs and 18 pairs, respectively). This suggests that the effect of APOE4 on brain networks is more pronounced in the context of Aβ positivity, possibly through accelerating amyloid-β deposition or independent neurodegenerative mechanisms, such as myelin maintenance abnormalities (Table 6).

**Table 4:** Impact of Aβ pathology in the context of APOE4 and gene-pathology interaction.

Differential connections (Pairs)		Within APOE4 + Group (Aβ + vs. Aβ-)	Within APOE4- Group (Aβ + vs. Aβ-)	APOE4 + Aβ + vs. APOE4-Aβ-	APOE4 + _Aβ- vs. APOE4-_Aβ+
SFC		6	8	136	28
DFC	Gau20	4	34	12	120
	Gau30	10	22	26	96
	Gau40	8	20	24	64
	Gau50	6	14	18	54
	Ham20	4	26	14	110
	Ham30	12	22	24	76
	Ham40	6	16	20	56
	Ham50	12	12	16	50

**Table 5:** Independent impact of APOE4 (Within Aβ+/− Groups).

Differential connections (Pairs)		Within Aβ + Group (APOE4 + vs. APOE4-)	Within Aβ- Group (APOE4 + vs. APOE4-)
SFC		82	70
DFC	Gau20	70	18
	Gau30	50	12
	Gau40	46	8
	Gau50	38	10
	Ham20	62	12
	Ham30	42	12
	Ham40	34	6
	Ham50	24	8

**Table 6:** SVM classification using fMRI dynamic graph parameters and kernel functions.

	ADNI2			ADNI 3		
Kernel function	Accuracy (%)	Sensitivity (%)	Specificity (%)	Accuracy (%)	Sensitivity (%)	Specificity (%)
Linear	70.19 ± 7.58	71.45 ± 11.95	69.65 ± 11.45	67.8 ± 5.3	66.81 ± 8.52	69.24 ± 9.76
Polynomial	69.06 ± 7.38	67.3 ± 11.19	72.07 ± 10.41	64.1 ± 5.12	63.16 ± 11.44	65 ± 11.05
RBF	76.7 ± 5.95	75.51 ± 6.12	78.27 ± 5.43	72.73 ± 5.86	73.33 ± 7.52	72.22 ± 5.86
Sigmoid	68.33 ± 8.13	68.2 ± 11.97	69.44 ± 12.89	65.66 ± 5.39	66.67 ± 8.2	64.91 ± 10.81

	ADNI Cohort Aβ subgroup			ADNI Cohort ApoE ε4 subgroup		
Kernel function	Accuracy (%)	Sensitivity (%)	Specificity (%)	Accuracy (%)	Sensitivity (%)	Specificity (%)
Linear	76.92 ± 3.78	77.78 ± 6.23	76.19 ± 6.15	76.92 ± 4.55	78.95 ± 5.97	75 ± 6.74
Polynomial	77.26 ± 4.28	72.33 ± 6.2	82.47 ± 6.77	78.25 ± 2.81	76.49 ± 9.16	79.61 ± 6.56
RBF	83.63 ± 4.02	83.25 ± 4.71	84.31 ± 6.9	85.16 ± 3.96	82.26 ± 6.1	86.14 ± 4.63
Sigmoid	75.57 ± 4.19	72.78 ± 6.72	78.53 ± 7.46	75.77 ± 3.97	71.22 ± 5.69	80.59 ± 6.54

Data are presented as mean ± standard deviation.

Supplementary Figure S1 (chord diagrams) visualized that short-window DFC (e.g., Gau20) detected early-stage differences in ventral attention and WM2/WM5, while long-window DFC (e.g., Ham50) and SFC highlighted late-stage alterations in default mode and visual networks.

### 3.3 Classification performance of cross-validated models across ADNI cohorts and subgroups

Prior to presenting cross-validation results, the selection of SVM kernel functions was guided by Supplementary Table S6. The Gaussian radial basis function (RBF) kernel achieved the highest classification accuracy (76.7% ± 5.95%) for differentiating SMC from CN across ADNI2 and ADNI3 cohorts, outperforming linear (70.19% ± 7.58%), polynomial (69.06% ± 7.38%), and sigmoid (68.33% ± 8.13%) kernels. This superiority motivated its use in subsequent analyses.

Classification results for full cohorts are summarized in Table 7. The dynamic graph theory model with RBF-SVM achieved the highest accuracy: 76.7% ± 5.95% (ADNI2) and 72.73% ± 5.86% (ADNI3), significantly surpassing static graph theory, traditional metrics, and clinical models (all *p* < 0.001).

**Table 7:** Classification results of different models for ADNI2 vs. ADNI3.

	ADNI2			ADNI3		
Classification features	Accuracy (%)	Sensitivity (%)	Specificity (%)	Accuracy (%)	Sensitivity (%)	Specificity (%)
Clinical	54.27 ± 8.6	55.54 ± 6.99	58.41 ± 5.41	52.17 ± 6.92	58.65 ± 6.04	49.18 ± 10.51
ReHo	60.59 ± 6.38	57.82 ± 9.69	64.14 ± 11.41	60.33 ± 6.05	56.93 ± 11.51	62.51 ± 11.71
ALFF	63.66 ± 6.41	62.71 ± 8.55	65.95 ± 10.08	60.28 ± 4.28	57.55 ± 8.61	64.08 ± 7.54
Static graph theory	69.37 ± 6.91	68.41 ± 6.46	71.23 ± 5.68	62.01 ± 5.72	61.89 ± 6.36	62.11 ± 6.86
Dynamic graph theory	76.7 ± 5.95	75.51 ± 6.12	78.27 ± 5.43	72.73 ± 5.86	73.33 ± 7.52	72.22 ± 5.86

Data are presented as mean ± standard deviation.

For Aβ and APOE ε4 subgroups (Table 1), the dynamic model achieved 83.63% ± 4.02% accuracy in Aβ + individuals and 85.16% ± 3.96% in APOE ε4 + subgroups. Notably, it showed a 0.15 AUC improvement in distinguishing SMC + from CI + within Aβ + subgroups, highlighting sensitivity to early pathology.

**Figure 5:**
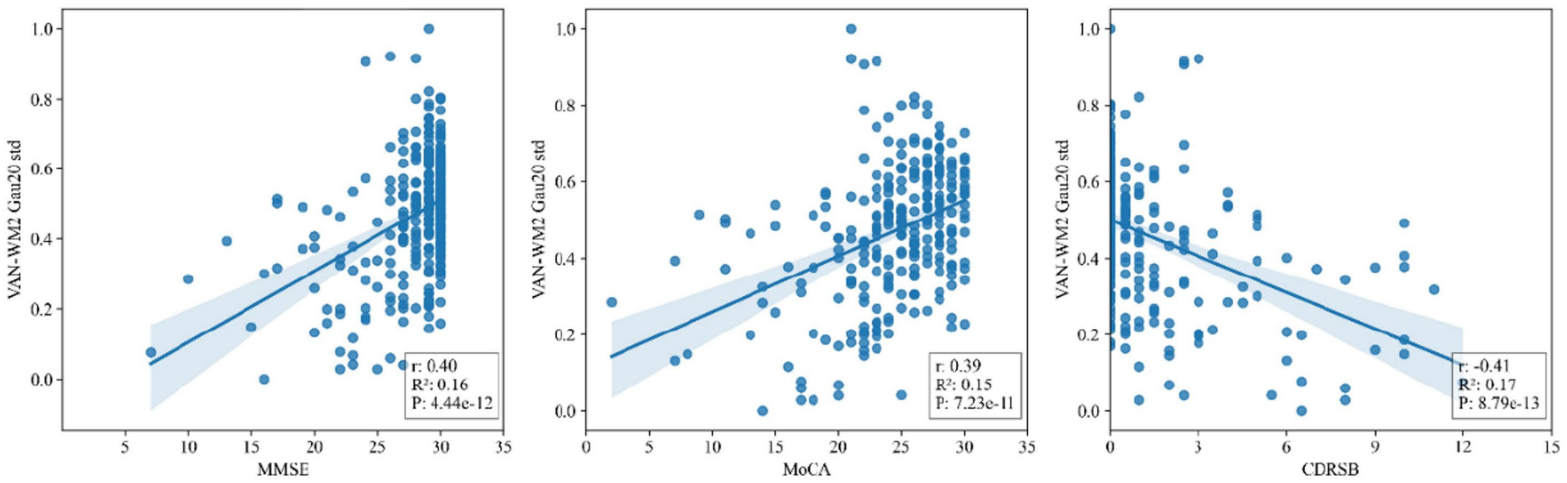
Scatter plots of correlations between salient dynamic connectivity features (VAN - WM2) and clinical cognitive scales (MMSE, MoCA, CDRSB). VAN - WM2 stands for Voxel - wise Alteration of Network - White Matter 2; MMSE for Mini - Mental State Examination; MoCA for Montreal Cognitive Assessment; CDRSB for Clinical Dementia Rating Scale - Sum of Boxes.

**Figure 6:**
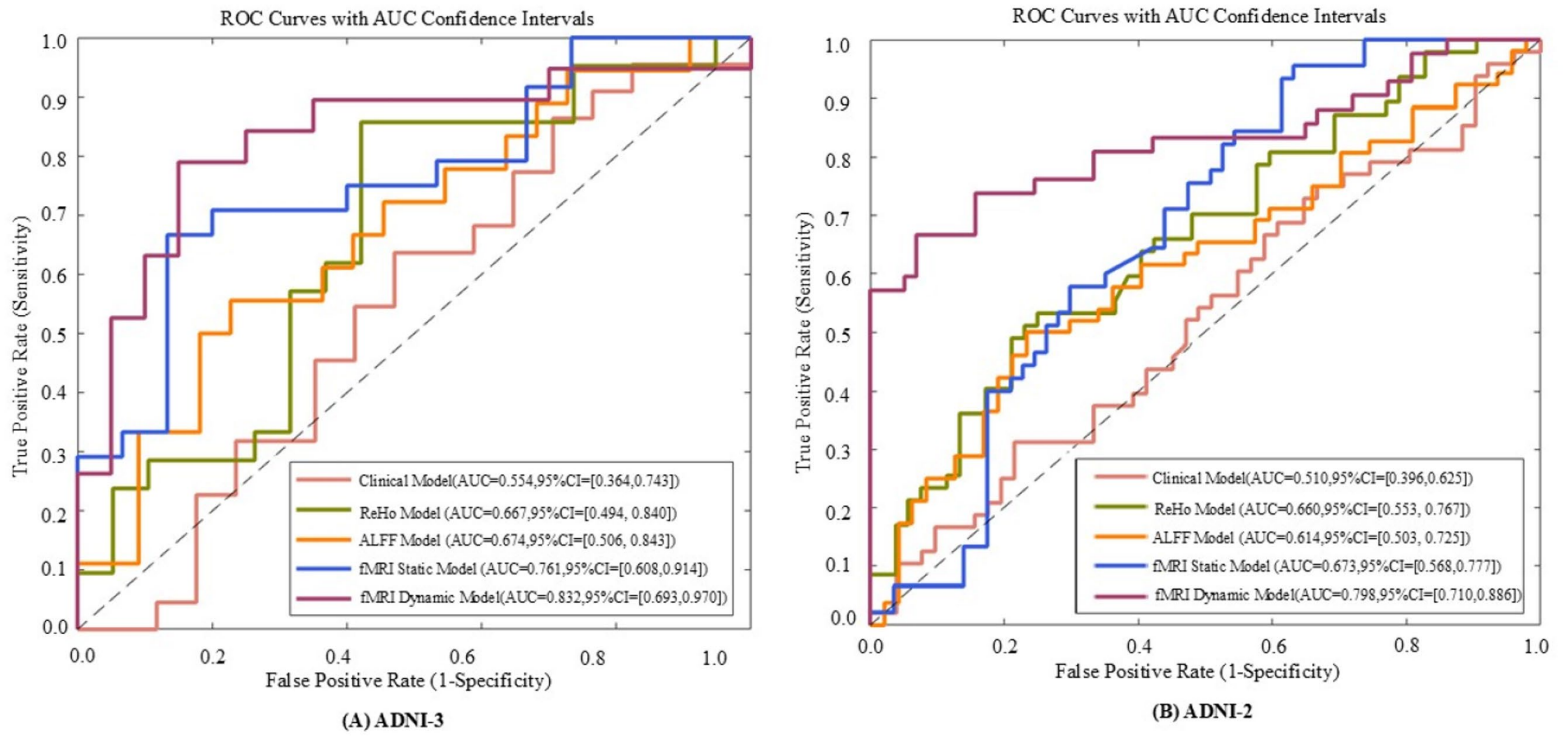
The receiver operating characteristic (ROC) curves of five models evaluated on ADNI - 3 **(A)** and ADNI - 2 **(B)** datasets. The five models include the fMRI dynamic graph theory model, fMRI static graph theory model, fMRI traditional metric ReHo model, fMRI traditional metric ALFF model, and clinical model.

**Figure 7:**
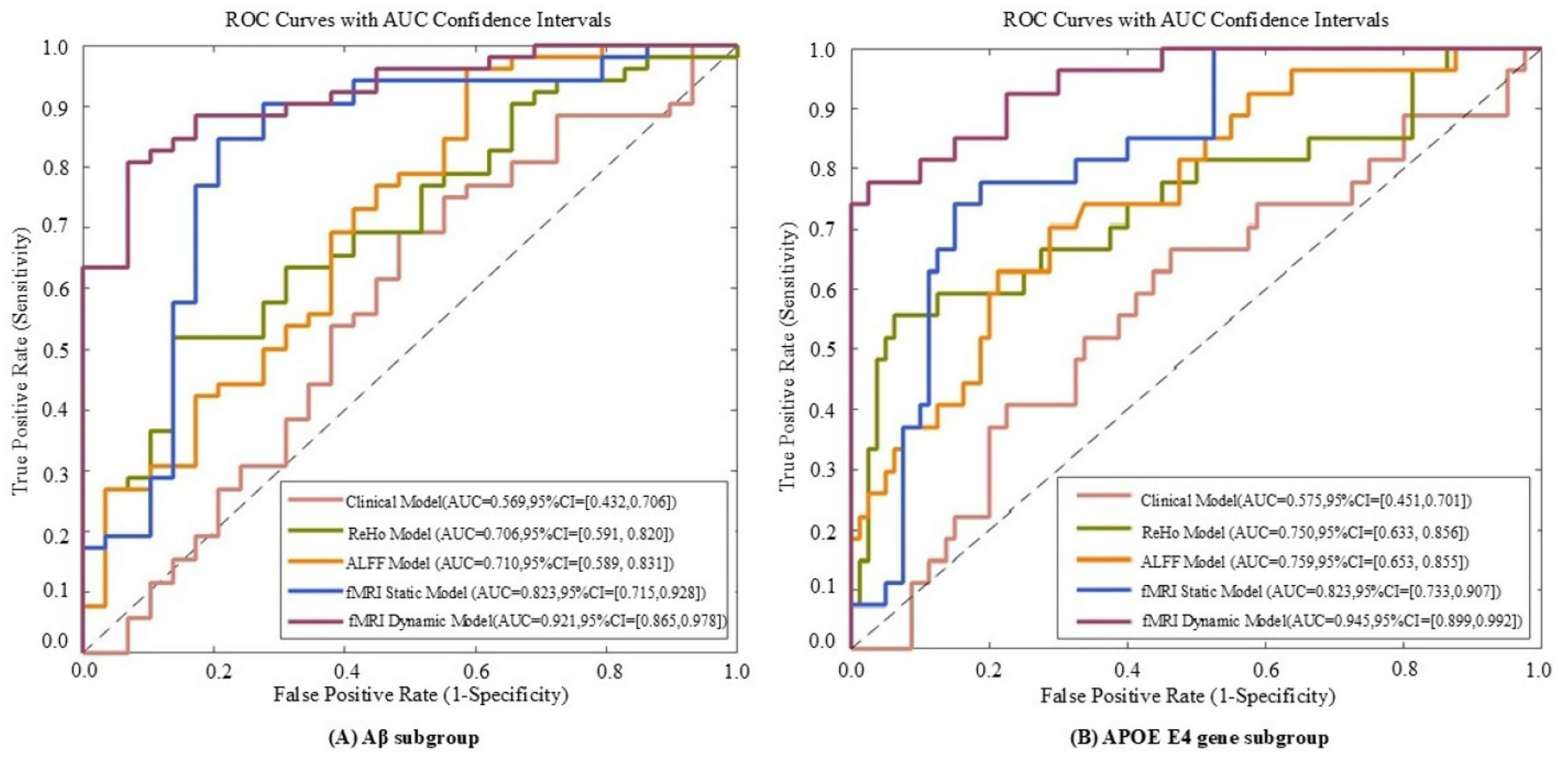
ROC curves of five models evaluated on Aβ subgroup **(A)** and APOE E4 gene subgroup **(B)** datasets. The five models include the fMRI dynamic graph theory model, fMRI static graph theory model, fMRI traditional metric ReHo model, fMRI traditional metric ALFF model, and clinical model.

Figure 5 presents scatter plots of correlations between the salient dynamic connectivity feature VAN-WM2 (Gau20 std) and clinical cognitive scales (MMSE, MoCA, CDRSB). The feature demonstrated significant positive correlations with MMSE (*r* = 0.40, *p* = 4.44 × 10^−12^) and MoCA (*r* = 0.39, *p* = 7.23 × 10^−11^), and a negative correlation with CDRSB (*r* = −0.41, *p* = 8.79 × 10^−13^), indicating that reduced dynamic connectivity in this white matter–gray matter interface is associated with worse cognitive performance (Figures 6, 7).

## 4 Discussion

### 4.1 Implications of demographic and clinical characteristics

The significant differences in APOE ε4 allele frequency and Aβ positivity between SMC and CI groups in both ADNI-2 and ADNI-3 cohorts (Table 2) confirm APOE ε4 as a major genetic risk factor for Alzheimer’s disease (AD) and highlight the association between Aβ pathology and cognitive decline. APOE ε4 carriers are more susceptible to Aβ deposition and synaptic damage, which accelerates the transition from subjective memory complaints to clinical cognitive impairment. The progressive decline in MMSE and CDR-SB scores suggests that the SMC stage already involves potential neurofunctional degradation, supporting the necessity of using DFC as an early monitoring indicator.

### 4.2 Stage-dependent features of DFC and SFC

DFC demonstrated superior cross-cohort stability compared to SFC (Supplementary Table S2), especially for short-window DFC (e.g., 20 TRs), with high similarity coefficients (0.862–0.933) indicating the conservation of neural dynamic fluctuations across different scanning conditions. Early-stage differences in DFC (30 connections in ventral attention and white matter networks) were observed before changes in SFC, suggesting that synaptic transmission efficiency or local field potential abnormalities may be among the earliest functional impairments in AD. In contrast, the marked increase in SFC differences in the late CI stage (118 connections involving the default mode network) aligns with neurodegenerative processes leading to brain network disintegration, confirming the hypothesis that “static connectivity reflects cumulative structural damage.

### 4.3 Interactions between genetics and pathology

The synergistic effect of APOE ε4 and Aβ was particularly evident in static connectivity, with 136 SFC differences in the default mode and limbic networks for APOE4 + Aβ + groups (Table 4; Supplementary Table S4), likely due to APOE4 promoting Aβ aggregation and neuroinflammation. Notably, APOE4 independently affected DFC in Aβ-negative individuals (70 connections in WM4/WM5), suggesting APOE4’s role in white matter dynamic stability through pathways unrelated to amyloid, such as myelin maintenance or axonal transport deficits, findings that align with Carrasco-Gómez et al. (2025). Further combining the results of Table 5, the network perturbation of APOE4 in the Aβ + population was significantly stronger than that in the Aβ- population (for example, the number of DFC differential connections between APOE4 + and APOE4- in the Aβ + group was 3.9 times that in the Aβ- group). This supports the hypothesis that “APOE4 mainly accelerates AD progression by enhancing the pathological effect of Aβ.” At the same time, the independent effect of APOE4 in the Aβ- group (such as 18 DFC differential connections) suggests that it may pre - disrupt the dynamic balance of brain networks through other pathways (such as lipid metabolism abnormalities), providing imaging evidence for “APOE4 being an independent risk factor for AD.

### 4.4 Clinical translation value of machine learning models

The high accuracy of the RBF-SVM model in distinguishing SMC from CN (76.7% ± 5.95%) is attributed to its ability to capture non-linear features of multi-window DFC. In Aβ + and APOE ε4 + subgroups, the model’s accuracy further increased to over 83%, with a 0.15 AUC improvement in early pathological conversion, indicating that DFC can serve as a biomarker for genetic-pathological stratification. The strong correlation between the VAN-WM2 dynamic connectivity feature and cognitive scales directly links functional network abnormalities to clinical phenotypes, providing a basis for interventions targeting the ventral attention-white matter interaction network in Figure 5.

### 4.5 Limitations and future directions

This study is based on cross-sectional data and requires longitudinal follow-up to validate the predictive value of DFC changes. Although cross-cohort scanning protocol differences were standardized, residual technical biases may still exist. Future research could integrate PET imaging (e.g., tau distribution) and single-cell sequencing to further elucidate the molecular and cellular mechanisms underlying DFC abnormalities, promoting its application in early AD diagnosis and treatment monitoring.

## 5 Conclusion

Dynamic functional connectivity (especially short-window DFC) can sensitively capture early functional abnormalities in gray-white matter networks in AD, with better stability and genetic-pathological associations than static indicators. The classification model combining machine learning provides a new paradigm for precise stratification and early intervention in AD, potentially becoming a core biomarker linking molecular pathology with clinical phenotype.

## Data Availability

Publicly available datasets were analyzed in this study. This data can be found at: https://adni.loni.usc.edu/.

## Footnotes

^1^ http://restfmri.net/forum/DPARSF

^2^ https://adni.loni.usc.edu/data-samples/adni-data/neuroimaging/mri/mri-scanner-protocols/

## The group member of the Alzheimer’s disease neuroimaging

ADNI is funded by the National Institute on Aging, the National Institute of Biomedical Imaging and Bioengineering, and through generous contributions from the following: AbbVie, Alzheimer’s Association; Alzheimer’s Drug Discovery Foundation; Araclon Biotech; BioClinica, Inc.; Biogen; Bristol-Myers Squibb Company; CereSpir, Inc.; Cogstate; Eisai Inc.; Elan Pharmaceuticals, Inc.; Eli Lilly and Company; EuroImmun; F. Hoffmann-La Roche Ltd. and its affiliated company Genentech, Inc.; Fujirebio; GE Healthcare; IXICO Ltd.; Janssen Alzheimer Immunotherapy Research and Development, LLC.; Johnson and Johnson Pharmaceutical Research and Development LLC.; Lumosity; Lundbeck; Merck and Co., Inc.; Meso Scale Diagnostics, LLC.; NeuroRx Research; Neurotrack Technologies; Novartis Pharmaceuticals Corporation; Pfizer Inc.; Piramal Imaging; Servier; Takeda Pharmaceutical Company; and Transition Therapeutics. The Canadian Institutes of Health Research is providing funds to support ADNI clinical sites in Canada. Private sector contributions are facilitated by the Foundation for the National Institutes of Health (www.fnih.org). The grantee organization is the Northern California Institute for Research and Education, and the study is coordinated by the Alzheimer’s Therapeutic Research Institute at the University of Southern California. ADNI data are disseminated by the Laboratory for Neuro Imaging at the University of Southern California.
