# Supplementary material for: Uncovering abnormal gray and white matter connectivity patterns in Alzheimer’s disease spectrum: a dynamic graph theory analysis for early detection
Source: Front Aging Neurosci. 2025 Jul 22;17:1589018. doi: 10.3389/fnagi.2025.1589018 (PMC12321872; doi:10.3389/fnagi.2025.1589018)
Supplement: Supplementary file 1 [file Table_1.docx]

Table S1: Summary of Window Types and Parameter Selection in Previous Sliding Window Analyses

| Sliding Window Type | Window Length (TRs) | Sliding Step Size | References |
| --- | --- | --- | --- |
| Hamming Window | 30 | 1 TR | (Lei et al., 2021)  (Muller and Virji-Babul, 2018) |
|  | 50 |  | (Cheng et al., 2021)  (Pedersen et al., 2018) |
|  | 40 |  |  |
| Gaussian Window | 20 |  | (Ma et al., 2019)  (Lin et al., 2018)  (Gan et al., 2021) (Sendi et al., 2021) |
|  | 22 |  | (Allen et al., 2014) (Bonkhoff et al., 2021) (Hidalgo de la Cruz et al., 2021) |

*Note:* TR (Repetition Time) denotes the interval between two consecutive radiofrequency pulse excitations during imaging, corresponding to the duration of one time point.

Table S1 summarizes the selection of window types and related parameters in previous sliding window analyses based on fMRI images. Gaussian window function reduces motion noise and demonstrates strong anti-interference capability in dynamic analysis, thus being widely applied in early sliding window studies. With methodological advancements, Hamming window function was found to effectively mitigate edge effects during window translation, further minimizing noise.

Studies indicate that excessively large window widths cause loss of dynamic information, while overly small windows introduce noise interference. Typically, window widths are set to 20–50 TRs (Repetition Time) based on actual imaging parameters. In this study, window widths of 30 TRs and 50 TRs were selected according to fMRI acquisition parameters. Given the subtle dynamic changes in the SCD (Subjective Cognitive Decline) stage, a 20 TRs window was additionally included. In summary, six sliding windows were employed for dynamic brain network construction: two window types (Gaussian and Hamming) with four window widths (20 TRs, 30 TRs, 40 TRs, 50 TRs). A uniform sliding step size of 1 TR was used to maximize utilization of dynamic information in fMRI time series.

Table S2: Stability Test of Static and Dynamic Functional Connectivity

| Cohort Comparison | Connectivity Type | Parameter | Similarity Coefficient (CN Groups) | Similarity Coefficient (SMC Groups) |
| --- | --- | --- | --- | --- |
|  |  |  | (ADNI2 Site 1 vs. Site 2 / ADNI3 Site 1 vs. Site 2 / ADNI2 vs. ADNI3) | (ADNI2 Site 1 vs. Site 2 / ADNI3 Site 1 vs. Site 2 / ADNI2 vs. ADNI3) |
| ADNI2 Internal | Static FC (SFC) | – | 0.769 | 0.785 |
|  | Dynamic FC (DFC) | Ham20 | 0.913 | 0.918 |
|  |  | Ham30 | 0.892 | 0.899 |
|  |  | Ham40 | 0.878 | 0.881 |
|  |  | Ham50 | 0.862 | 0.868 |
|  |  | Gau20 | 0.922 | 0.925 |
|  |  | Gau30 | 0.902 | 0.908 |
|  |  | Gau40 | 0.882 | 0.894 |
|  |  | Gau50 | 0.876 | 0.882 |
| ADNI3 Internal | Static FC (SFC) | – | 0.769 | 0.785 |
|  | Dynamic FC (DFC) | Ham20 | 0.913 | 0.918 |
|  |  | Ham30 | 0.892 | 0.899 |
|  |  | Ham40 | 0.878 | 0.881 |
|  |  | Ham50 | 0.862 | 0.868 |
|  |  | Gau20 | 0.922 | 0.925 |
|  |  | Gau30 | 0.902 | 0.908 |
|  |  | Gau40 | 0.882 | 0.894 |
|  |  | Gau50 | 0.876 | 0.882 |
| ADNI2 vs. ADNI3 | Static FC (SFC) | – | 0.777 | 0.783 |
|  | Dynamic FC (DFC) | Ham20 | 0.921 | 0.927 |
|  |  | Ham30 | 0.901 | 0.907 |
|  |  | Ham40 | 0.886 | 0.892 |
|  |  | Ham50 | 0.872 | 0.878 |
|  |  | Gau20 | 0.933 | 0.939 |
|  |  | Gau30 | 0.912 | 0.918 |
|  |  | Gau40 | 0.891 | 0.897 |
|  |  | Gau50 | 0.884 | 0.891 |

Note: SFC was defined as the average connection strength across the entire time series. DFC was defined as the mean standard deviation across all links in the first dimension (i.e., averaged over all functional connections).

Table S3: Subset-Level Static vs. DFC in ADNI 2

| Differential Connections (Pairs) | | ADNI-2 | | | | | |
| --- | --- | --- | --- | --- | --- | --- | --- |
|  |  | Site3 | | | Site4 | | |
|  |  | SMC vs. CN | SMC vs. CI | CN vs. CI | SMC vs. CN | SMC vs. CI | CN vs. CI |
| SFC |  | 3 | 45 | 20 | 0 | 76 | 54 |
| DFC | Ham20 | 30 | 8 | 14 | 0 | 18 | 20 |
|  | Ham30 | 34 | 10 | 38 | 0 | 22 | 34 |
|  | Ham40 | 28 | 12 | 48 | 2 | 18 | 32 |
|  | Ham50 | 26 | 18 | 54 | 2 | 18 | 28 |
|  | Gau20 | 22 | 6 | 10 | 2 | 20 | 20 |
|  | Gau30 | 32 | 10 | 32 | 0 | 24 | 28 |
|  | Gau40 | 32 | 12 | 40 | 0 | 16 | 30 |
|  | Gau50 | 26 | 14 | 52 | 0 | 14 | 30 |

Note: Ham20 refers to a Hamming window with a size of 20 seconds. Gau20 refers to a Gauss window with a size of 20 seconds. Similarly, Gau30, Gau40, and Gau50 represent Gauss windows with sizes of 30 seconds, 40 seconds, and 50 seconds accordingly. Site 3and Site 4 differ in the number of fMRI time points, where Site 1 includes data with 200 time points, and Site 2 with 9600 time points.

Table S4: Connectivity Strength Analysis of Static and Dynamic Brain Networks (Gaussian vs. Hamming Windows, TR=20,30,40,50) in SMC vs. CN Groups

| Source subnet | Target subnet | Static | Gau20 | Ham20 | Gau30 | Ham30 | Gau40 | Ham40 | Gau50 | Ham50 |
| --- | --- | --- | --- | --- | --- | --- | --- | --- | --- | --- |
| Visual | Visual | 0.968 | 0.823 | 0.817 | 0.864 | 0.881 | 0.876 | 0.851 | 0.848 | 0.826 |
| Somatomotor | Somatomotor | 0.921 | 0.824 | 0.827 | 0.892 | 0.92 | 0.913 | 0.895 | 0.884 | 0.873 |
| Dorsal Attention | Dorsal Attention | 0.936 | 0.906 | 0.901 | 0.957 | 0.99 | 0.99 | 0.975 | 0.977 | 0.965 |
| Ventral Attention | Ventral Attention | 0.889 | 0.584 | 0.587 | 0.67 | 0.7 | 0.712 | 0.709 | 0.712 | 0.706 |
| Limbic | Limbic | 0.834 | 0.793 | 0.776 | 0.827 | 0.846 | 0.849 | 0.833 | 0.834 | 0.818 |
| Frontoparietal Control | Frontoparietal Control | 0.835 | 0.851 | 0.839 | 0.889 | 0.905 | 0.904 | 0.878 | 0.879 | 0.856 |
| Default Mode | Default Mode | 0.784 | 0.85 | 0.827 | 0.861 | 0.871 | 0.865 | 0.84 | 0.842 | 0.823 |
| WM01 | WM01 | 0.514 | 0.302 | 0.299 | 0.313 | 0.314 | 0.324 | 0.318 | 0.33 | 0.322 |
| WM02 | WM02 | 1 | 0.41 | 0.434 | 0.533 | 0.571 | 0.59 | 0.601 | 0.607 | 0.615 |
| WM03 | WM03 | 0.709 | 0.852 | 0.849 | 0.883 | 0.888 | 0.886 | 0.847 | 0.85 | 0.817 |
| WM04 | WM04 | 0 | 0 | 0 | 0 | 0 | 0 | 0 | 0 | 0 |
| WM05 | WM05 | 0.464 | 0.399 | 0.372 | 0.406 | 0.409 | 0.419 | 0.423 | 0.435 | 0.438 |
| WM06 | WM06 | 0.689 | 0.017 | 0.023 | 0.171 | 0.218 | 0.251 | 0.284 | 0.291 | 0.315 |
| WM07 | WM07 | 0.908 | 0.115 | 0.12 | 0.164 | 0.175 | 0.189 | 0.203 | 0.21 | 0.224 |
| WM08 | WM08 | 0.876 | 0.615 | 0.634 | 0.716 | 0.754 | 0.759 | 0.751 | 0.746 | 0.736 |
| WM09 | WM09 | 0.996 | 1 | 1 | 1 | 1 | 0.975 | 0.929 | 0.925 | 0.887 |
| WM10 | WM10 | 0.942 | 0.833 | 0.845 | 0.939 | 0.996 | 1 | 1 | 1 | 1 |
| WM11 | WM11 | 0.776 | 0.807 | 0.799 | 0.84 | 0.854 | 0.855 | 0.832 | 0.83 | 0.809 |
| WM12 | WM12 | 0.573 | 0.481 | 0.477 | 0.523 | 0.535 | 0.545 | 0.539 | 0.548 | 0.542 |

*Note:* Static Functional Connectivity (SFC) was defined as the average connection strength across the entire time series. For Dynamic Functional Connectivity (DFC), we first computed the standard deviation of connection strength for each functional link across time (capturing temporal variability). Then, DFC was defined as the mean standard deviation across all links in the first dimension (i.e., averaged over all functional connections)

Figure S1:Chord Diagrams Illustrating Functional Connectivity Variations
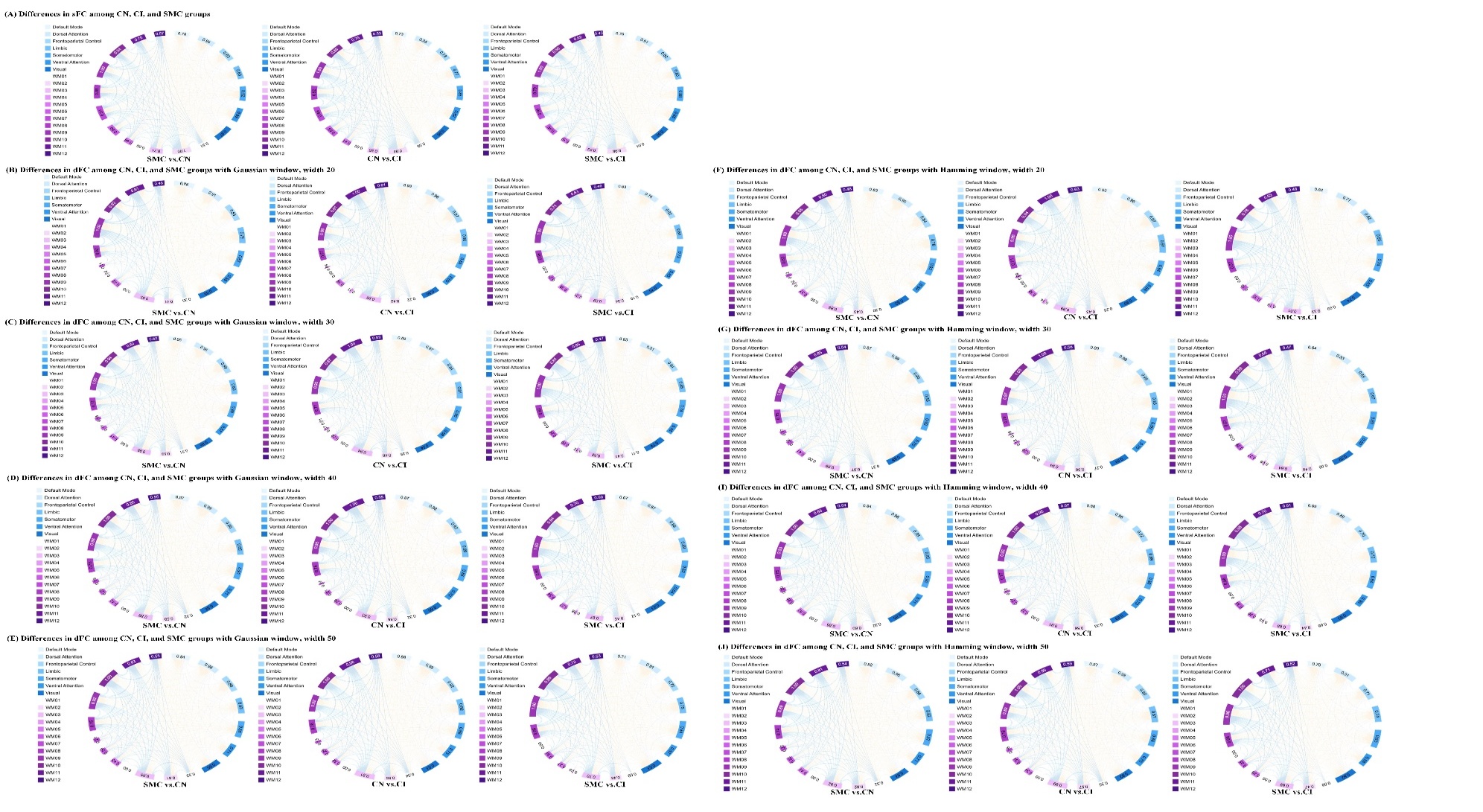


**Reference**

Allen, E.A., Damaraju, E., Plis, S.M., et al. (2014) Tracking whole-brain connectivity dynamics in the resting state. *Cerebral Cortex (New York, N.Y.: 1991)*, 24 (3): 663–676.

Bonkhoff, A.K., Schirmer, M.D., Bretzner, M., et al. (2021) Abnormal dynamic functional connectivity is linked to recovery after acute ischemic stroke. *Human Brain Mapping*, 42 (7): 2278–2291.

Cheng, Y., Zhang, G., Zhang, X., et al. (2021) Identification of minimal hepatic encephalopathy based on dynamic functional connectivity. *Brain Imaging and Behavior*, 15 (5): 2637–2645.

Gan, C., Ma, K., Wang, L., et al. (2021) Dynamic functional connectivity changes in Parkinson’s disease patients with REM sleep behavior disorder. *Brain Research*, 1764: 147477.

Hidalgo de la Cruz, M., Valsasina, P., Sangalli, F., et al. (2021) Dynamic Functional Connectivity in the Main Clinical Phenotypes of Multiple Sclerosis. *Brain Connectivity*, 11 (8): 678–690.

Lei, T., Liao, X., Chen, X., et al. (2021) *Development of Large-Scale Brain Network Dynamics in Children*. p. 2021.03.03.433828.

Lin, S., Vavasour, I., Kosaka, B., et al. (2018) Education, and the balance between dynamic and stationary functional connectivity jointly support executive functions in relapsing–remitting multiple sclerosis. *Human Brain Mapping*, 39 (12): 5039–5049.

Ma, W.-Y., Yao, Q., Hu, G.-J., et al. (2019) Dysfunctional Dynamics of Intra- and Inter-network Connectivity in Dementia With Lewy Bodies. *Frontiers in Neurology*, 10: 1265.

Muller, A.M. and Virji-Babul, N. (2018) Stuck in a State of Inattention? Functional Hyperconnectivity as an Indicator of Disturbed Intrinsic Brain Dynamics in Adolescents With Concussion: A Pilot Study. *ASN neuro*, 10: 1759091417753802.

Pedersen, M., Zalesky, A., Omidvarnia, A., et al. (2018) Multilayer network switching rate predicts brain performance. *Proceedings of the National Academy of Sciences*, 115 (52): 13376–13381.

Sendi, M.S.E., Zendehrouh, E., Ellis, C.A., et al. (2021) Aberrant Dynamic Functional Connectivity of Default Mode Network in Schizophrenia and Links to Symptom Severity. *Frontiers in Neural Circuits*, 15: 649417.
